# Cephalic Venous Aneurysm and Intravascular Papillary Endothelial Hyperplasia in a Blood Donor

**DOI:** 10.14740/jmc5325

**Published:** 2026-06-03

**Authors:** Christodoulos Chatzigrigoriadis, Georgios-Eleftherios Anagnostopoulos, Dimitra Koumoundourou, Eriselda Mamullari, Kiriaki Zikou, Georgios Marios Stergiopoulos, Panagis Galiatsatos, Stavros Kakkos

**Affiliations:** a School of Medicine, University of Patras, 26504 Patras, Greece; b Department of Pathology and Cytopathology, University General Hospital of Patras, 26504 Patras, Greece; c Department of Radiology, University General Hospital of Patras, 26504 Patras, Greece; d Department of Internal Medicine, Georgetown University, Washington, DC 20007, USA; e Department of Internal Medicine, Johns Hopkins University School of Medicine, Baltimore, MD 21224, USA; f Department of Vascular Surgery, University of Patras Medical School, 26504 Patras, Greece

**Keywords:** Aneurysm, Thrombosis, Thrombophlebitis, Vascular malformations, Phlebotomy, Venipuncture, Intravascular papillary endothelial hyperplasia, Masson hemangioma

## Abstract

Venous aneurysms (VAs) and pseudoaneurysms (VPAs) are uncommon vascular lesions with variable etiology and location. We report a 52-year-old male blood donor who developed thrombophlebitis presenting as a right antecubital mass after multiple venipunctures. Initial duplex ultrasound revealed a saccular dilation of the right cephalic vein (15.6 × 7.8 × 25.5 mm) with thrombus, as well as thrombosis of the right brachial vein. The patient was treated with anticoagulation, followed by definitive surgical management. Histopathological examination revealed a VA, as well as intravascular papillary endothelial hyperplasia. A scoping review of PubMed and Scopus databases identified seven similar cases of upper extremity VAs or VPAs related to venipuncture or peripheral venous cannulation. To our knowledge, this is an unusual case of a right cephalic VA and intravascular papillary endothelial hyperplasia secondary to venipuncture. Current knowledge on this uncommon disease entity is summarized.

## Introduction

An aneurysm is defined as an abnormal dilation of the vessel lumen covered by all layers of the vascular wall, while a pseudoaneurysm is an extravascular hematoma connected with the vessel lumen without an endothelial layer after a contained rupture of the vascular wall [1–4]. Although the definition of aneurysmal dilation in veins is unclear, a 50% or 100% increase in diameter over normal has been proposed [1, 2, 4–9].

The rarity of venous aneurysms (VAs) and venous pseudoaneurysms (VPAs) compared to equivalent arterial pathologies is probably related to the lower intraluminal blood pressure [10]. Osler published the first case of a VPA in 1913, which involved the popliteal vein [11]. Since then, multiple reports have been published in various locations, particularly in the deep venous system of the lower extremities [2, 3, 6, 7, 12]. VA/VPAs can be either congenital or acquired and may also be spontaneous or secondary to a specific trigger [1, 6, 7, 12]. A minority of VA/VPAs are observed in the superficial veins of the upper extremities, and only a few of them are associated with venipuncture or peripheral intravenous lines [2, 3, 12].

In this manuscript, we report an unusual case of cephalic VA with histopathological evidence of intravascular papillary endothelial hyperplasia (IPEH) in the setting of multiple venipunctures in a 52-year-old male blood donor. In addition, a scoping review of upper extremity VA/VPAs secondary to venipunctures and peripheral vein cannulation was conducted, with seven similar cases being isolated [5, 10, 13–16]. To our knowledge, this case represents a unique combination of superficial VA, deep venous thrombosis, and IPEH following venipuncture.

## Case Report

We report the case of a 52-year-old Caucasian male who presented with upper extremity pain and a right antecubital fossa mass. The mass was reportedly present for 18 months, but for 3 days before presentation, the patient developed a self-resolving episode of pain at the mass site, which prompted him to seek care. The patient reported 20 blood donations (six for platelets using a 17-gauge needle and 14 for whole blood using a 16-gauge needle, according to blood bank records) that had been drawn from his upper limb superficial venous system, including the site of the mass. Other past medical history included pneumonia in 2020, not otherwise specified; removal of multiple benign skin nevi for cosmetic concerns; and complete excision of the basal cell carcinoma before 8 months. He denied regular use of any medications and reported no known allergies. Regarding his social history, the patient reported prior tobacco use (12 pack-years), which was discontinued 30 years ago, and he worked as an aircraft engineer.

**Figure 1:**
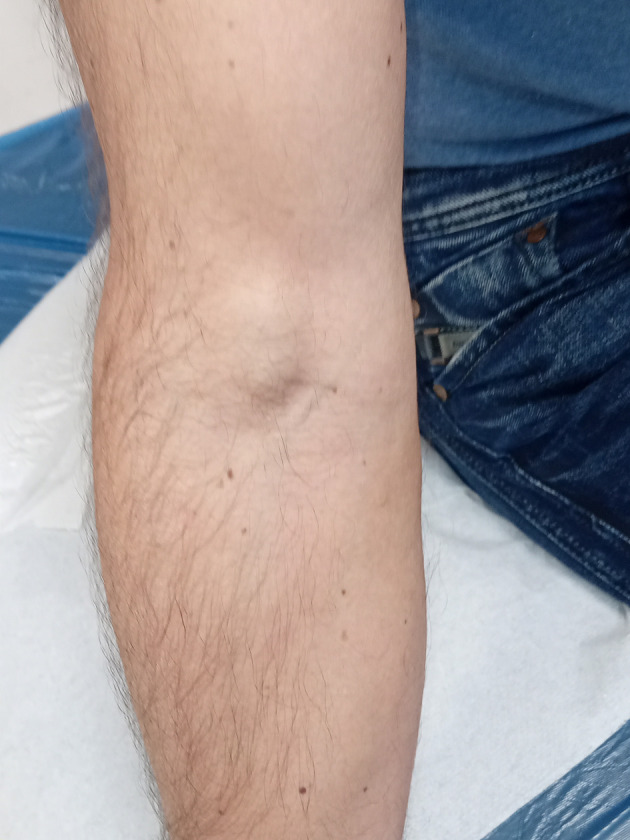
Physical examination of the patient revealed a 25-mm soft, non-pulsatile mass in the right antecubital fossa. Tenderness and the absence of a bruit were also noted.

Physical examination revealed a soft and non-pulsatile mass (25 mm in length), with tenderness on the right antecubital fossa without a bruit (Fig. 1). When the arm was moved from an elevated to a dependent position, a significant enlargement of the lesion was observed. There was no evidence of erythema, raised temperature, lymphadenopathy, arterial or neurological impairment of the right arm.

**Figure 2:**
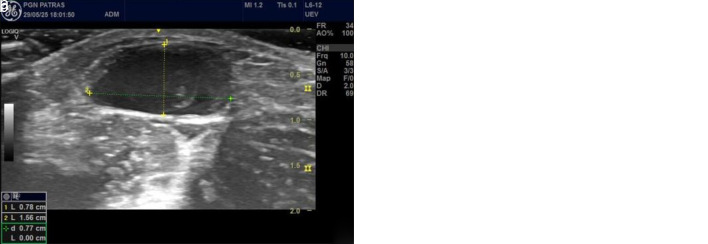
Venous duplex ultrasound of the right cephalic vein revealed saccular dilatation measuring 15.6 × 7.8 × 25.5 mm with hyperechogenic intraluminal material consistent with superficial venous aneurysm and thrombosis.

At this point, the origin of the mass was strongly suspected to be vascular, with the working diagnosis being a VA; therefore, a venous duplex ultrasound (US) of the arm was performed in the dependent position (Fig. 2), revealing a saccular dilation (15.6 × 7.8 × 25.5 mm) of the right cephalic vein. Additionally, hyperechogenic material was present within the saccular lesion as well as the underlying brachial vein, indicating superficial vein thrombosis along with deep venous thrombosis of the upper extremity. Blood flow was maintained in the cephalic vein but was absent in the brachial vein. Laboratory evaluation, including complete blood count, coagulation panel, and basic metabolic panel, was unremarkable.

**Figure 3:**
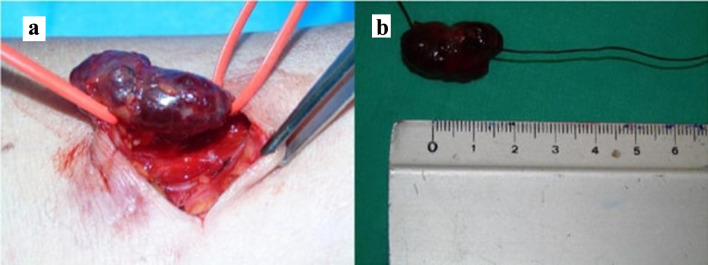
(a) Intraoperative image showing ligation of the afferent and efferent venous branches, and excision of the cephalic vein aneurysm. (b) Surgical specimen of the resected cephalic vein aneurysm.

**Figure 4:**
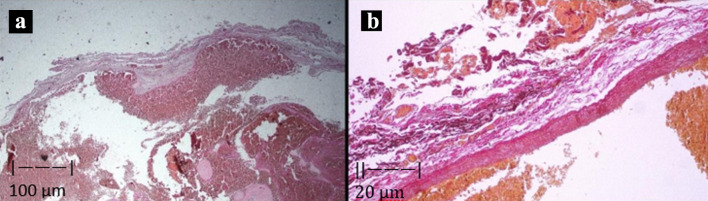
(a) Microphotograph depicting a transverse section of the vessel with dilatation of the lumen as well as disorganization (markedly reduced) of the muscle layer and the elastic fiber network. Hematoxylin and eosin stain was used at × 25 magnification. (b) The network of elastic fibers is disrupted and replaced. Collagen fibers replace smooth muscle and elastic fibers consistent with endophlebosclerosis. Van-Gieson stain was used at × 100 magnification.

**Figure 5:**
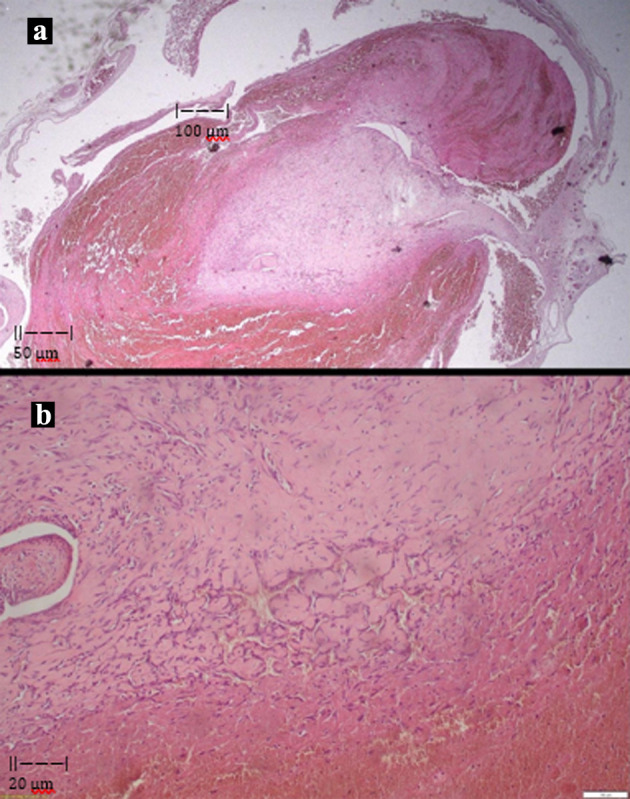
(a) Microphotograph depicting a transverse section in hematoxylin and eosin stain (H&E) of the vessel with dilatation of the lumen, as well as the presence of intravascular papillary endothelial hyperplasia. Hematoxylin and eosin stain was used at × 25 magnification. (b) A focus of intravascular papillary endothelial hyperplasia is shown, composed of numerous papillary structures lined by plump endothelial cells. Hematoxylin and eosin stain was used at × 100 magnification.

Anticoagulation with apixaban (5 mg twice daily) was administered for 3 months for the patient’s deep venous thrombosis, while the use of elastic stockings was deemed unnecessary. At the follow-up appointment, gradual resolution of the pain in the upper extremity was reported. In addition, there was no evidence of thrombi, and the lesion’s size on duplex US was unchanged. Discontinuation of apixaban and surgical management were recommended. Excision of the VA along with ligation of the afferent and efferent cephalic vein under local anesthesia was performed (Fig. 3a). Gross examination revealed an elongated hemorrhagic mass measuring 22 mm in length and 10 mm in maximal diameter (Fig. 3b). Microscopically, a medium-sized vein was recognized with dilation of the lumen and indistinct boundaries between the vascular layers. A reduced smooth muscle layer and disorganized elastic fibers, replaced by collagen fibers (endophlebosclerosis), were noted in the pathology report after the use of hematoxylin and eosin stain and Van-Gieson stain (Fig. 4). The presence of IPEH was also confirmed by the identification of proliferating reactive endothelial cells forming numerous papillary structures typically lined by a single layer of plump endothelial cells after the use of hematoxylin and eosin stain (Fig. 5). Two weeks later the patient’s sutures were removed without complications.

## Discussion

**Figure 6:**
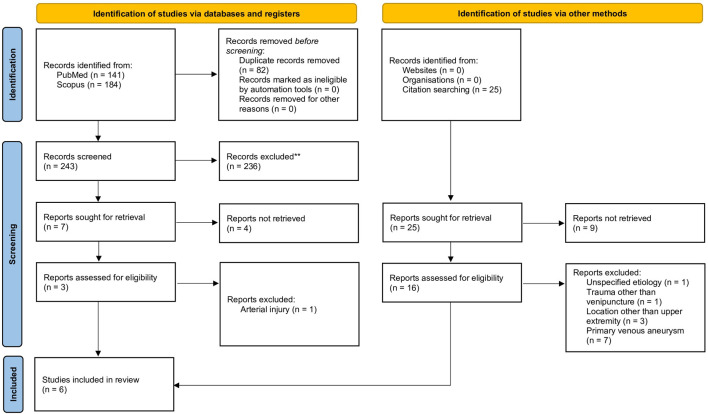
The selection process of suitable cases for the literature review is presented in a flow diagram.

A search was conducted in June 2025 using PubMed and Scopus to identify cases of upper extremity VA/VPAs following venipuncture or peripheral vein cannulation. The search strategy was: (vein* OR venous*) AND (pseudoaneurysm* OR aneurysm*) AND (iatrogenic* OR venipuncture* OR phlebotomy* OR traumatic* OR catheterization*) AND (cephalic* OR basilic* OR superficial*). An extensive search of the included articles for relevant publications was also performed. CC and SK finalized the search terms and the research criteria, and CC was responsible for the review process (Fig. 6). The inclusion criteria were publications in the English language describing VA/VPAs of the upper extremities in association with blood sampling, blood donation, and the placement of a peripheral intravenous line. Exclusion criteria included: publications in languages other than English; arterial aneurysms/pseudoaneurysms; VA/VPAs of the lower extremities; spontaneous VA/VPAs; VA/VPAs with arteriovenous fistula; and traumatic VA/VPAs with a different mechanism. The following information was extracted and presented: past medical history of anticoagulation, age, sex, trigger, onset after exposure, clinical presentation, size, diagnostic imaging, treatment, follow-up, and histopathology.

A total of 350 articles (141 from PubMed and 184 from Scopus) were identified, of which 82 duplicates were removed, and 236 were excluded following review of the title and abstract. Additionally, the full texts of 13 articles were not retrieved. Therefore, full-text screening was conducted on 19 articles; ultimately, six articles with seven patients were selected for inclusion. Finally, eight cases are presented (including the present) with upper extremity VA/VPA after venipuncture or peripheral vein cannulation (Table 1).

**Table 1:** Reported Cases in the Literature, Including the Present Case, Regarding Upper-Extremity Venous Aneurysms and Pseudoaneurysms Secondary to Venipuncture or Peripheral Vein Cannulation

Author/year	No.	Anticoagulation	Age (years)/sex/vein	Etiology/onset	Clinical presentation	Size (mm)	Imaging	Treatment	Follow-up	Histopathology
Niimi et al, 2017 [16]	1	No	58/female/right basilic vein	Blood sampling/3 years	Non-pulsatile antecubital mass with compression of the lateral antebrachial cutaneous nerve	24 × 19	Duplex US, MRI: VPA with thrombus	Persistent thrombus despite anticoagulation Successful surgical resection with ligation	2 years; no recurrence	VPA: dilatation with a thickened intima and media
	2	No	56/female/left basilic vein	Venipuncture/2 months	Non-pulsatile antecubital mass	23 × 17	Duplex US, MRI: VPA	Successful surgical resection	6 months; no recurrence	VPA: dilatation with thrombus
Ward et al, 2009 [14]	3	Yes	64/male/right basilic vein	Venipuncture/immediately	Non-pulsatile antecubital mass	40 × 30 position-dependent	B-mode and duplex US: 43 × 33 × 20 mm cavity with low-pressure swirling flow	Unsuccessful compression Successful surgical resection with ligation	n/a	VPA: lack of endothelial lining
Lotfi et al, 2007 [10]	4	No	43/male/left median antecubital vein	Venipuncture for blood donation/immediately	Non-pulsatile, tender antecubital mass	30 position-dependent	US: 30 × 15 mm cystic lesion with thrombosis Doppler analysis: swirling flow	Successful surgical resection and repair of the puncture site	n/a	VPA: lack of vascular wall with granulation tissue and thrombosis
Chakraborty et al, 1999 [13]	5	Yes	57/female/left antecubital vein	Venipuncture/immediately	Non-pulsatile, tender antecubital mass	45	US, venography: compressible VPA	Successful embolization	6 weeks; no recurrence	n/a
Debnath et al, 2007 [5]	6	No	45/female/right median cubital vein	Peripheral intravenous line/few months	Non-pulsatile, non-tender antecubital mass	20 × 30	Duplex US: VA	Successful surgical resection with ligation	n/a	VA: preservation of three layers, thickened media, and congested lumen
Perler, 1990 [15]	7	No	39/female/unspecified	Peripheral intravenous line/immediately	Non-pulsatile, non-tender wrist mass	35 × 15	Venous Doppler analysis; normal Venography: VA	Surgical resection with ligation	n/a	VA
Our case	8	No	52/male/right cephalic vein	Venipuncture for blood donation/unspecified	Non-pulsatile, tender antecubital mass	25 position-dependent	Duplex US: saccular dilation (15.6 × 7.8 × 25.5 mm) with thrombus	Anticoagulation and surgical resection with ligation	2 weeks; no recurrence	VA: dilation of the vascular lumen with disorganization of the vascular wall; endophlebosclerosis and IPEH

IPEH: intravascular papillary endothelial hyperplasia; MRI: magnetic resonance imaging; US: ultrasound; VA: venous aneurysm; VPA: venous pseudoaneurysm.

Anticoagulant use before diagnosis was reported in 2/8 cases. The mean age was 52 years (range: 39–64 years), and 5/8 patients were female. Regarding the clinical presentation, all cases (8/8) presented with a non-pulsatile antecubital mass. Tenderness was observed in 3/8 patients, and neuropathy was reported in 2/8 patients. Lesion size ranged from 2 to 4.5 cm, while position-dependent changes were observed in 3/8 cases.

US-based imaging was the primary diagnostic technique in all reported cases (8/8). The lesions displayed variable dimensions. Important features, including thrombus (3/8) and swirling flow (2/8), were reported. Additional imaging modalities, such as magnetic resonance imaging (MRI) (2/8) and venography (2/8), were used occasionally to further characterize the lesions.

Management was predominantly surgical; in 7/8 cases, resection and ligation of the affected vein were performed. Embolization was successfully performed in a single case. Conservative measures, such as anticoagulation (2/8) and compression therapy (1/8), were less effective; anticoagulation resolved the thrombus in one case and failed in another, requiring surgical intervention. Follow-up periods ranged from 2 weeks to 2 years, with no recurrences or procedure-related complications reported.

Histopathology was available for 7/8 cases. VPAs (4/8) exhibited venous dilation with thickened intima and media with disruption of the vascular wall by granulation tissue and thrombosis, as well as the lack of normal endothelial lining. In contrast, true VAs (3/8) demonstrated preserved wall architecture with thickened media as well as congestion, thrombosis, and IPEH.

We report a rare case of right cephalic VA secondary to repeated venipunctures for blood donation with microscopic evidence of IPEH, also known as Masson’s hemangioma. It was complicated by symptomatic thrombosis extending to the right brachial vein, which was shown by US. Treatment included initial anticoagulation followed by definitive surgical resection with confirmatory pathology results.

Our case represents a superficial upper extremity VA affecting the right cephalic vein, which is a rare vascular lesion of unknown prevalence. VA/VPAs may be congenital or acquired, with a variable age of presentation and equal distribution in both sexes [1, 3, 7, 17]. Sometimes, they are associated with an arteriovenous fistula [1, 7]. Regarding their location, they are grouped in the head and neck, chest, abdomen, and extremities (either superficial or deep veins) [1, 3, 6, 7, 12]. The most common location is the deep venous system of the lower extremities (especially the popliteal vein) [3, 7].

The mechanism of this right cephalic VA appears to be related to repeated venipunctures for blood donation, with an unclear latency period. The development of IPEH in this biopsy suggests a repair process of vascular trauma. Only a few cases of iatrogenic VA/VPAs were identified in the literature review; such lesions may be underdiagnosed or misdiagnosed as idiopathic [5, 7, 10, 13–16]. VA/VPAs are classified as primary or secondary based on their etiology [3, 4, 7, 9, 12, 17]. Connective tissue disorders, coagulopathy, venous hypertension, trauma, surgery, venipuncture, vein cannulation, intravenous drug use, venous graft, and arteriovenous fistula precede the diagnosis of secondary VA/VPA with an interval of a few weeks to more than a decade [1, 2, 12, 14, 17–19]. The pathogenesis of VA/VPAs is related to genetic and environmental factors, as well as reactive changes [1, 2, 5]. Underlying pathological processes include 1) congenital weakness of the vascular wall; 2) endophlebohypertrophy (intimal hypertrophy) and endophlebosclerosis (replacement of muscular wall and elastic fibers by collagen-rich connective tissue) due to turbulent flow in bifurcation or intersection sites; 3) degenerative changes in response to mechanical stress; and 4) repeated microtrauma [1, 5, 7–9, 19]. Thrombosis is often present, probably due to blood stasis [1, 3, 4, 12]. Although the causal relationship is currently unclear, it is hypothesized that IPEH represents a persistent repair process of pre-existing vascular lesions, trauma, or thrombosis [20]. However, its biological behavior resembles a benign endothelial tumor with aggressive features [21].

The patient developed an acute episode of tenderness in a pre-existing lesion of the antecubital fossa. At the same time, a soft, non-pulsatile tumor without bruit was noted. The absence of pulsatile blood flow and the position-dependent size strongly pointed to venous pathology. Deep vein thrombosis of the adjacent brachial vein was also identified during the investigation. The clinical presentation of VA/VPA ranges from asymptomatic lesions to severe complications, depending on the site, size, rate of growth, and neighboring structures [1, 3, 4]. A superficial lesion of the upper extremities typically presents as a soft, non-pulsatile, and compressible mass [1, 2, 4, 9]. Clinically relevant features specific to these lesions include enlargement during exercise and Valsalva maneuver, while elevation of the affected extremity decreases their size [1, 2, 9, 16, 17]. Sudden painful expansion, cosmetic disfigurement, and nerve entrapment are commonly observed [1, 2, 14, 16, 17]. Life-threatening complications are unlikely to occur with head and neck, chest, and upper extremity (especially superficial) VA/VPAs [3, 4, 12]. However, intra-abdominal VA/VPAs are often complicated by rupture and gastrointestinal hemorrhage, while lower extremity (especially deep) VA/VPAs are highly associated with thrombosis and pulmonary embolism [3, 8, 12, 22].

Duplex US was performed to investigate the patient’s mass, which was clinically suspected to be vascular. A saccular dilation of the right cephalic vein with hyperechogenic material in the lumen of the right cephalic and brachial veins was detected. The radiologic diagnosis of superficial VA complicated by thrombophlebitis was confirmed histopathologically after surgical excision. It is well established that imaging studies confirm the clinical suspicion of VA/VPA and rule out other entities, such as lymphadenopathy, soft tissue tumors, hygromas, hematomas, ganglion cysts, and other vascular pathologies [1–3, 9, 16]. They also provide details regarding the location, presence of concomitant thrombosis, and feeding vessels, thus guiding the treatment plan [3, 14]. Duplex or triplex US are first-line tests for the investigation and assessment of acral or head and neck VA/VPAs [1, 3, 4, 7, 9, 12, 17, 19]. Although computed tomography angiography (CTA) and magnetic resonance angiography (MRA) are often used in the management of superficial VA/VPA, their typical indications include the diagnosis of chest and abdominal lesions as well as the preoperative assessment of deep venous lesions [1, 3, 4, 7, 12, 17, 19, 22]. Notably, MRA is preferred in patients with impaired renal function to avoid exposure to nephrotoxic agents. Although conventional angiography has been used as a diagnostic tool, its current utility is limited to interventional treatment [4, 12, 13]. A definite diagnosis between VA and VPA can only be established through histopathological examination of the excised lesion [1, 5, 10, 13, 14].

Treating this superficial VA was preferred over observation to alleviate symptoms and reduce the risk of pulmonary embolism. Anticoagulants were used for managing superficial thrombophlebitis and deep venous thrombosis. Additionally, saccular morphology, superficial thrombophlebitis, and deep venous thrombosis clearly indicated the need for surgical intervention in our patient; simple excision and ligation were deemed sufficient, rather than vascular repair. Limited evidence exists regarding the treatment of upper extremity VA/VPAs, which depends on clinical presentation and patient preferences [1, 4, 9, 12]. Since they tend to be benign, observation is reasonable for small, stable, and asymptomatic lesions of the upper extremities with fusiform morphology, provided lack of thromboembolism or cosmetic concerns [1, 3, 4, 7–9, 12]. However, a recent systematic review of 40 patients with upper extremity VA revealed that most (95%) underwent surgery [4]. Surgery is often preferred for cosmetic reasons due to its low risk of perioperative complications and recurrence [1–4, 14]. Moreover, excision of the lesion with ligation of its feeding vessels is usually sufficient for superficial VA/VPAs, but deep VA/VPAs often require additional revascularization techniques [4, 8, 9, 12]. Endovascular methods such as stenting, coil embolization, catheter-directed thrombolysis, sclerotherapy, and thrombin injection are alternative options in patients with comorbidities or for lesions that are difficult to access [9, 12, 13, 17]. US-guided compression is a minimally invasive technique, although it may be ineffective in anticoagulated patients or with chronic lesions [1, 10, 13, 14]. Surgery is generally necessary for VA/VPAs located in the abdomen or the deep veins of the lower limbs, whereas a watch-and-wait approach is usually preferred for head, neck, and thoracic VA/VPAs due to their lower complication rates [3, 7, 10, 12]. Medical management with anticoagulants for 3–6 months is recommended for thrombosed VA/VPAs to reduce the risk of thromboembolism, regardless of their location [1, 3, 7, 8].

This study presents a case report of cephalic VA following phlebotomy and reviews seven upper extremity VA/VPAs with a similar etiology. Given the limited data, it was not feasible to follow the Preferred Reporting Items for Systematic Reviews and Meta-Analyses (PRISMA) guidelines for statistical analysis and risk of bias assessment. Thus, a scoping review based on case reports instead of a systematic review was conducted, which is the major limitation of this study. Further research with observational studies is essential to clarify the relationship between phlebotomy and venous pathologies and to estimate the risk of simple procedures in healthcare.

### Learning points

Iatrogenic VA/VPAs in upper extremities remain a rare and probably underdiagnosed entity. Position-dependent size changes and absence of a pulse are essential clinical features. Disfigurement, nerve compression, thrombophlebitis, and thrombosis are the main concerns. US is the first-line diagnostic test. Medical management involves observation in selected cases or anticoagulation for thromboembolic complications. However, surgery is the preferred option in most cases; surgical excision with ligation is the most widely used technique for superficial lesions in the upper extremities. This case builds on the current evidence around VA/VPAs in the setting of prior venipuncture. It presents an interesting constellation of clinical, imaging, and histopathological features, including cephalic vein thrombophlebitis, brachial vein thrombosis, and histopathological evidence of IPEH. Clinicians, nurses, and patients should be aware of this rare complication of venipuncture.

## Data Availability

The authors declare that data supporting the findings of this study are available within the article.

## Abbreviations

CTA: computed tomography angiography
IPEH: intravascular papillary endothelial hyperplasia
MRA: magnetic resonance angiography
MRI: magnetic resonance imaging
PRISMA: Preferred Reporting Items for Systematic Reviews and Meta-Analyses
US: ultrasound
VA: venous aneurysm
VPA: venous pseudoaneurysm
